# Engineering Behavior and Characteristics of Wood Ash and Sugarcane Bagasse Ash

**DOI:** 10.3390/ma8105353

**Published:** 2015-10-12

**Authors:** Francisco Grau, Hyunwook Choo, Jong Wan Hu, Jongwon Jung

**Affiliations:** 1 Department of Civil and Environmental Engineering, Louisiana State University, Baton Rouge, LA 70803, USA; fgrau@alumni.lsu.edu; 2 School of Civil, Environmental and Architectural Engineering, Korea University, Seoul 136701, Korea; choohw@gmail.com; 3 Department of Civil and Environmental Engineering, Incheon National University, Incheon 22012, Korea; jongp24@incheon.ac.kr; 4 Incheon Disaster Prevention Research Center, Incheon National University, Incheon 406110, Korea

**Keywords:** biomass, wood ash, sugarcane bagasse ash, characterization

## Abstract

Biomasses are organic materials that are derived from any living or recently-living structure. Plenty of biomasses are produced nationwide. Biomasses are mostly combusted and usually discarded or disposed of without treatment as biomass ashes, which include wood and sugarcane bagasse ashes. Thus, recycling or treatment of biomass ashes leads to utilizing the natural materials as an economical and environmental alternative. This study is intended to provide an environmental solution for uncontrolled disposal of biomass ashes by way of recycling the biomass ash and replacing the soils in geotechnical engineering projects. Therefore, in this study, characteristic tests of wood and sugarcane bagasse ashes that are considered the most common biomass ashes are conducted. The test of chemical compositions of biomass ashes is conducted using energy dispersive X-ray spectroscopy (EDS), and Scanning Electron Microscope (SEM), and heavy metal analysis is also conducted. Engineering behaviors including hydraulic conductivity, constrained modulus and shear modulus are examined. Also, coal fly ash Class C is used in this study for comparison with biomass ashes, and Ottawa 20/30 sands containing biomass ashes are examined to identify the soil replacement effect of biomass ashes. The results show that the particle sizes of biomass ashes are halfway between coal fly ash Class C and Ottawa 20/30 sand, and biomass ashes consist of a heterogeneous mixture of different particle sizes and shapes. Also, all heavy metal concentrations were found to be below the US Environmental Protection Agency (EPA) maximum limit. Hydraulic conductivity values of Ottawa 20/30 sand decrease significantly when replacing them with only 1%–2% of biomass ashes. While both the constrained modulus and shear modulus of biomass ashes are lower than Ottawa 20/30 sand, those of mixtures containing up to 10% biomass ashes are little affected by replacing the soils with biomass ashes.

## 1. Introduction

Nowadays, environmental concerns and an interest in reducing construction costs have led to using some recycled materials instead of conventional materials for engineering projects, resulting in favorable outcomes in terms of both economical and technical aspects. For example, the engineering properties of coal fly ash Class C have been studied to examine the possibility of recycling for engineering projects including highway constructions [1], concrete mixtures [2] and soil stabilization [3,4]. Considering the fact that geotechnical projects generally require a large quantity of materials, reuse of biomass ashes as a new recycled material in geotechnical projects will surely be attractive.

Biomasses, including plants, are organic materials that are derived from any living or recently-living structure. Biomass has been used as an agriculture, forest, and energy resource, such as for biofuels, as a source of industrial heat for the forestry and paper industries, and for ethanol and biodiesel [5]. Biomasses are mostly combusted and then typically discarded or disposed of without treatment. Thus, recycling or treatment of biomass ashes leads to the utilization of natural materials as an economical and environmental alternative. In 2012, about 474 million dry tons of biomass materials were estimated to be created in the United States from forest and agricultural residues [6]. Forests residues constitute approximately 231 million dry tons of this total, with wood as the most abundant biomass material in the United States. A remaining 243 million dry tons are divided into multiple agricultural crops including sugarcane bagasse [6]. Combustion of biomass produces biomass ashes. The total amount of biomass ashes obtained from combustion is variable depending on the type of material and combustion process. Total annual biomass ashes may range from 4.6–27 million dry tons [7]. Both wood ash and sugarcane bagasse ash account for the greater portion of the biomass ashes [6].

The use of wood ash and sugarcane bagasse ash as a pozzolanic material in concrete mixtures [8,9] and the use of wood ash as highway pavement and subgrade reinforcement material [10,11] have been studied in an attempt to increase the strength and stiffness of the materials [12]. However, the study of geotechnical properties of biomass ashes lags far behind. Thus, this study aims at understanding the geotechnical properties and engineering behaviors of biomass ashes, thereby providing economical materials by replacing the soils with biomass ashes as well as reducing waste. Also, coal fly ash Class C is used to compare the properties with biomass ashes.

## 2. Literature Review

### 2.1. Biomass Classification

**Figure 1:**
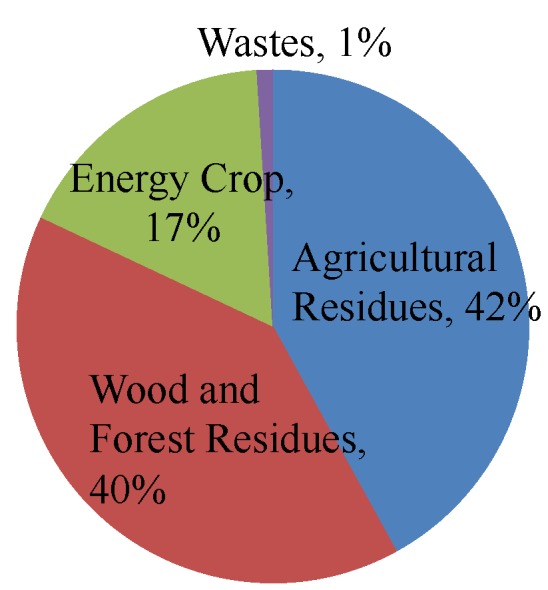
Biomass classification [13].

Biomass is divided into three main groups (Figure 1): (1) forest biomass and wood waste resources include the residues of harvested wood, branches, and leaves on the ground that account for about one-third of the trees, corresponding to 40% of the total biomass available, and (2) agricultural biomass and waste resources consist of plants and animal-based residual materials which all constitute recycling materials after harvest crops including roots, leaves, fruits, stems and other parts of the plants. Incinerating these residues produces the ashes that constitute 42% of the total biomass in the United States. The ashes are illegally dumped or used as soil fertilizers and (3) biomass energy crops are dedicated to the plants grown at low economic cost and used merely for energy which accounts for 17% of the biomass. Table 1 shows the annual biomass available and the ashes obtained after burning the biomass that depends on the type of biomass and combustion process.

The crops used in this research are wood and sugarcane because forest biomass and wood waste account for the greater portion of biomass materials in the United States and reached 231 million tons in 2012; which is expected to increase further to 286 million tons in 2017 (See Table 1) [6,7]. Although the total wood ashes can hardly be calculated, the total estimated annual wood ashes is estimated at 0.9–4.9 million dry tons in 2012 which is expected to increase to up to 6 million tons in 2017 [7]. Also, agricultural biomass and waste reached 243 million tons in 2012 which is expected to further increase to 292 million tons in 2017 (See Table 1) [6,7]. Among agricultural biomass, sugarcane is considered the most important and the leading farm product [14,15].

**Table 1:** Annual biomass availability [6,7].

Type of Biomass	Biomass in 2012 (Million Dry Tons)	Estimated Biomass in 2017 (Million Dry Tons)	Percentage of Ash Obtained (%)
Forest biomass and wood waste	231	286	0.4–2.1
Agricultural biomass and waste	243	292	1.5–9.1

### 2.2. Physical and Chemical Properties of Biomass 

Table 2 shows the average particle size (*d*_50_), bulk density and specific surface area of biomass ashes and coal fly ash Class C. Wood ash and coal fly ash indicate similar average particle size while sugarcane bagasse ash is notably smaller than others. In case of bulk density, sugarcane bagasse ash values are smaller than wood ash, and coal fly ash Class C. Wood ash shows a relatively high specific surface area that provides good absorption [16]. The specific surface area presented by sugarcane bagasse ash is also higher than coal fly ash. Table 3 shows the chemical compositions of wood ash, sugarcane bagasse ash and coal fly ash Class C. All of them include a large quantity of *SiO_2_* (31.8%–85.5%).

**Table 2:** Average particle diameter, bulk density, and specific surface areas of ashes [9,17,18,19,20,21,22,23,24].

Materials	Average Particle Diameter (*d_50_*) (mm)	Bulk Density (kg/m³)	Specific Surface Area (m²/kg)
Wood ash	0.230	663—977	4200—100,600
Sugarcane bagasse ash	0.023	410—590	900—943
Coal fly ash Class C	0.208	750—800	392—471

**Table 3:** Chemical composition of wood ash, sugarcane bagasse ash, and coal fly ash Class C [25,26,27].

Constituents	Wood Ash	Sugarcane Bagasse Ash	Coal Fly Ash Class C
SiO_2_	31.8	85.5	40
Al_2_O_3_	28	5.3	17
Fe_2_O_3_	2.34	1.3	6
CaO	10.53	2.1	24
MgO	9.32	1.1	5
SO_3_	-	-	3
Na_2_O	6.5	-	-
K_2_O	10.38	3.5	-

## 3. Experimental Section

### 3.1. Materials

Wood and sugarcane bagasse ashes which are the most common biomass ashes are used in this study as biomass ashes. Also, Ottawa 20/30 sand and coal fly ash Class C is used for comparison with biomass ashes in the tests. Laboratory tests are performed to identify the characteristics. Wood ashes are transported from a wood manufacturing company, RoyOMartin in Louisiana, which are obtained after incinerating the wood residues of southern yellow pine trees. Sugarcane bagasse ashes are collected from Alma Plantation Sugarcane Mill in Lakeland, Louisiana after burning the leaves, branches, stems and other residual parts. Biomass ashes used in this study are bottom ashes. Both companies have used the combustion technique of biomass in ASTM E1755; (1) the sample is taken from 0–250 °C in 1 h, (2) it is kept constant at 250 °C for at least 1 more hour. Then, (3) temperature is increased from 250–575 °C in 30 min, (4) it is kept constant at 575 °C for at least 2 h, (5) Sample is taken out of the furnace and Mass is measured. (6) Finally, the process above is repeated two times. So, in total, the sample night remain for 9 h inside the furnace.

### 3.2. Sample Preparation 

In order to understand geotechnical behavior of mixed materials by replacing the soils with biomass ashes, Ottawa 20/30 sand-biomass ash mixtures are used in this study. Ottawa 20/30 sands are used as the large particle host materials, while biomass ashes are added to Ottawa 20/30 sands. Ottawa 20/30 sands are replaced with the biomass ashes the same in weight to keep a constant total dry weight. A variety of dry weight ratios (0.5%–10%) of mixtures that represent the dry weight of Ottawa 20/30 sand and the weight of biomass ashes are prepared for laboratory tests.

### 3.3. Experimental Methods

*Characterization.* Sieve analysis (ASTM C117) and hydrometer tests (ASTM D422) are conducted with Ottawa 20/30 sand, wood ash, sugarcane bagasse ash and coal fly ash Class C. Specific surface area (ASTM C1069-09) is measured with wood ash, sugarcane bagasse ash and coal fly ash Class C using specific surface area analyzer (Gemini VII 2390, Micrameritics, Norcross, GA, USA). Specific gravity tests are conducted with a 500 mL water picnometer and a vacuum pump to extract air bubbles in pore (ASTM D854). pHs of materials are measured using a benchtop pH meter (Thermo Scientific Orion, 2 stars, Fisher Scientific, Pittsburgh, PA, USA). Ten grams of material are collected and mixed with 10 mL of water in a centrifuge tube. Then, the tubes are placed in the centrifuge before spinning those samples to extract the water from the pores of the materials and to measure the pH (ASTM D4972). Scanning Electron Microscope (SEM) (JEOL, Peabody, MA, USA) is used to examine the microstructure of the materials. Samples are fixed with a two-layer adhesive carbon and aluminum pin studs to be placed in the SEM adapter. Since biomass ashes are not conductive materials, the samples need to be coated with a conductive layer of gold to prevent the charging of the specimen. After coating, the pin studs are inserted into the specimen stage so that the image processing can be taken with the Scanning Electron Microscope. X-ray diffraction (XRD), Empyrean manufactured by Panalytical (Panalytical, Westborough, MA, USA), is used to analyze the qualitative and quantitative chemical composition of biomass ashes and coal fly ash Class C. Also, heavy metal concentrations in biomass ashes were explored by total metal analysis using inductively coupled plasma optical emission spectrometry (ICP-OES) (PerkinElmer, Waltham, MA, USA). Samples digested in highly acidic (pH < 2) solution at 90 °C were prepared for ICP-OES analysis without boiling. Chemically bound metals within the sample were released into solution (US EPA, 1996). The mixture of the acid solution and sample was filtered to remove fine particulates and the filtrate was analyzed using a Perkin Elmer Optima 7300 DV ICP-OES (PerkinElmer).

*Hydraulic Conductivity.* Constant head tests (ASTM D2434) are conducted using Ottawa 20/30 sand. Diameter of specimen (*D* = 10.2 cm), height of specimen (*L* = 11.6 cm), head difference (Δ*H* = 25 cm) and void ratio (*e* = 0.56~0.70) are controlled. Falling head tests are conducted with biomass ashes (ASTM D5084). The diameter of specimen (*D* = 7.62 cm), height of specimen (*L* = 14.61 cm), initial head difference (Δ*H* = 36.7 cm) and void ratio (*e* = 1.20~3.47) are controlled. Also, Ottawa 20/30 sand-biomass ash mixtures including 0.5%, 1%, 1.5% and 4% biomass ashes are used at a similar initial void ratio (*e* = 0.571~0.654) to Ottawa 20/30 sand with constant head tests.

**Figure 2:**
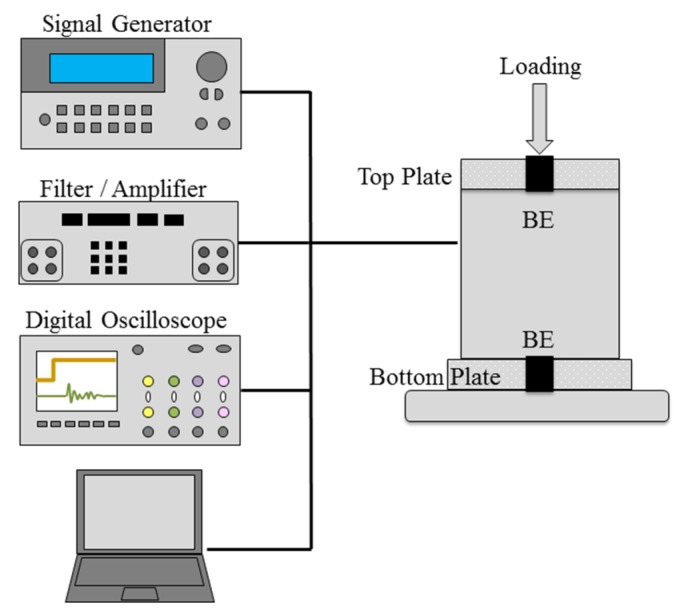
Schematic drawing of shear wave velocity measurement setup during consolidation.

*Consolidation.* One-dimensional consolidation tests are conducted with biomass ashes, Ottawa 20/30 sands, and Ottawa 20/30 sand-biomass ash mixtures with a variety of dry weight ratios (2%, 4%, 6%, 8%, 10%) (ASTM D2435). Test samples are set up in a stainless steel chamber that consists of two top and bottom plates with drainage ports to prevent segregation (Figure 2). Diameter of specimen (*D* = 6.4 cm) and height of specimen (*L* = 4.0 cm) are controlled. Initial void ratios of Ottawa 20/30 sand (*e* = 0.632–0.674) and biomass ashes (*e* = 1.55–2.77) are controlled. Initial void ratios of Ottawa 20/30 sand-biomass ash mixtures are similar to Ottawa 20/30 sand (*e* = 0.632–0.674). Loading (12–192 kPa), unloading (192–24 kPa) and reloading (24–1536 kPa) with the same incremental ratio equivalent to 1 are applied to the specimen using a GeoJac automated loading system made by GeoTAC (Houston, TX, USA), which is instrumented with a 2000 load-pound capacity load sensor at the bottom of the frame and a direct current displacement transducer (DCDT) on top. This loading system is controlled using the software Sigma-1 ICON (GeoTAC) for Windows. The loading schedule is set up on GeoJac at the beginning of the tests and loading interval for specimens was determined to be 12 hours according to several preliminary tests that confirm the end of the primary consolidation of these materials for the proposed loading steps. (Note: In the preliminary tests, all primary consolidations of biomass ashes are completed within 4~6 h).

*Shear Wave Velocity.* While one-dimensional consolidation tests are conducted, the shear wave velocity is measured for every load increment using bender elements set inside the modified oedometer cell, which allows the measurement of the shear wave velocity as a function of increasing consolidation stress [28] (Figure 2). The shear wave which travels through the soil specimen is generated by a function generator (33210A, Agilient, Santa Clara, CA, USA), with a square wave of frequency = 20 Hz and amplitude = 10 V, which is connected to the source bender element. The bender element that acts as the signal receiver is connected to the filter amplifier (3364, Krohn-Hite, Brockton, MA, USA), which in turn is connected to the digital oscilloscope (DSO6014A, Agilent, Santa Clara, CA, USA). A total of 1024 signals are stacked to reduce the influence of uncorrelated noise. The travel time of the shear wave is determined using the digitized signal as recorded by the oscilloscope [24], while tip-to-tip distance (the distance from the tip of the source bender element to the tip of the receiver bender element) is used as the travel distance [28,29].

## 4. Experimental Results

The characteristics of Ottawa 20/30 sand and biomass ashes are summarized. Hydraulic conductivity, consolidation, and shear wave velocity are following.

### 4.1. Basic Properties of Biomass Ashes

**Figure 3:**
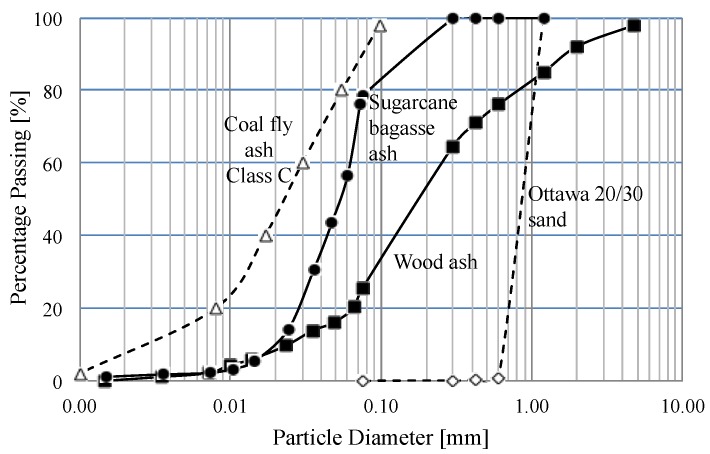
Particle size distribution of Ottawa 20/30 sand, biomass ashes and coal fly ash Class C.

*Particle Size Distribution.* Figure 3 shows the results of the sieve analysis and hydrometer test of each material which are repeated twice. The results show that particle size distributions of biomass ashes are somewhere between Ottawa 20/30 sand and coal fly ash Class C (Figure 3). Wood ash and sugarcane bagasse ash include 25.4% and 78% fine particles, which are less than 75-μm (No. 200 sieve) in size. Table 4 shows all coefficients obtained from the particle size distribution in Figure 3.

**Table 4:** Properties of wood ash, sugarcane bagasse ash and Ottawa 20/30 sand.

Test	Ottawa 20/30 Sand	Coal Fly Ash Class C	Wood Ash	Sugarcane Bagasse Ash
*d*_10_ (mm)	0.650	0.003	0.025	0.020
*d*_30_ (mm)	0.750	0.013	0.090	0.046
*d*_50_ (mm)	0.870	0.022	0.180	0.055
*d*_60_ (mm)	0.900	0.030	0.260	0.063
C*_u_*	1.38	10.00	10.40	3.15
C*_c_*	0.96	1.88	1.25	1.68
Specific surface area (m^2^/kg)		411~423	12,025~14,025	922~932
G*_s_*	2.65	2.58	2.41	2.34
pH	4.01	-	12.57	8.65

*d*_10_ = grain diameter at 10% passing, *d*_30_ = grain diameter at 30% passing, *d*_50_ = grain diameter at 50% passing, *d*_60_ = grain diameter at 60% passing, *C_u_* = Coefficient of uniformity, *C_c_* = Coefficient of curvature, *G_s_* = Specific gravity.

*Specific Gravity, Atterberg Tests, pH, Specific Surface.* Specific gravities of wood ash (*G_s_* = 2.41) and sugarcane bagasse ash (*G_s_* = 2.34) and coal fly ash Class C (*G_s_* = 2.58) are smaller than Ottawa 20/30 sand (*G_s_* = 2.65) (Table 4). Acidity results indicate that wood ash (pH = 12.57) and sugarcane bagasse ash (pH = 8.65) have high alkalinity while Ottawa 20/30 sand has acidity (pH = 4.01) (Table 4). While coal fly ash Class C shows *LL* = 22.5 that is consistent with previous study (Note. *LL* is liquid limit) [30], and both wood and sugarcane bagasse ashes used in this study do not show plasticity. Wood ash has a quite higher specific surface area (12,025~14,025 m^2^/kg) than other sugarcane bagasse ash (922~932m^2^/kg) and coal fly ash Class C (411~423 m^2^/kg) that is consistent with previous study as outlined in Table 2. According to Ban and Ramli (2011), high values of specific surface area of wood ashes are attributable to a high degree of irregularity in particle shape and porosity of the surface [31].

**Figure 4:**
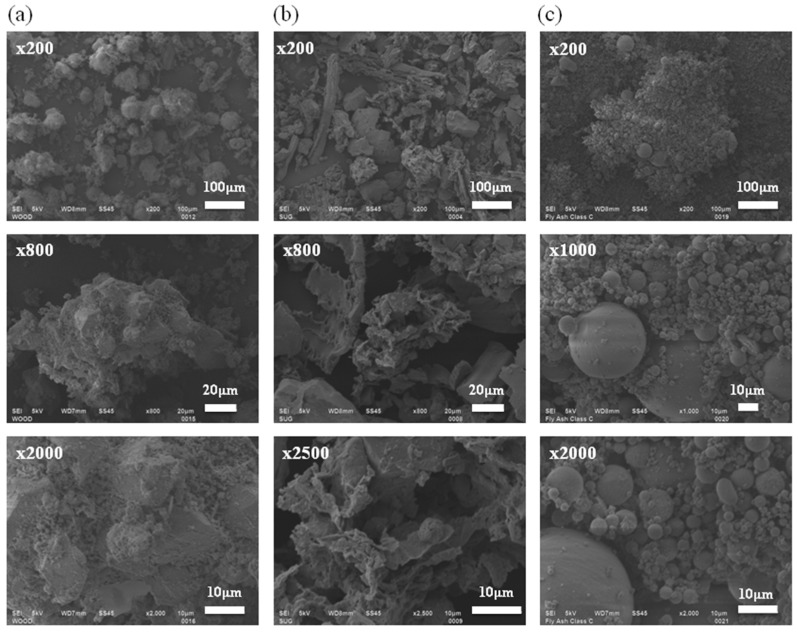
SEM images of ashes: (**a**) wood ash, (**b**) sugarcane bagasse ash, (**c**) coal fly ash class C.

*SEM Images. *All photomicrographs of ashes used in this study show a heterogeneous mixture in different sizes and shapes. Particle morphologies of ashes are shown in Figure 4. SEM images of wood ash show various particle sizes (10–200 μm) and the sub-angular shapes (Figure 4a) that are consistent with the results of particle size distribution in Figure 3. In previous studies, Naik and Etiégni also observed the irregularly-shaped inorganic particles in wood ashes [17,18]. In the case of sugarcane bagasse ash, their shapes are mainly sub-angular and they have low sphericity; however, they have a scattered particle shape and size to generalize the common shape. The particle size of the sugarcane bagasse ash observed in this study varies from 10–300 μm which is consistent with the results of particle size distribution (Figure 3 and Figure 4b). These SEM results are consistent with the results of previous studies that indicate a mixture of particles with rough surfaces, high porosity and large surface areas in sugarcane bagasse ashes through SEM micrographs [9]. Both wood and sugarcane bagasse ashes have porous structures that can explain the lower *G_s_* and higher specific surface areas of both ashes than Ottawa 20/30 sand (Table 4). Most coal fly ash Class C consists of solid spheres and the diameter appears to be smaller than biomass ashes (Figure 3).

*Chemical Composition.* The results of XRD analysis are consistent with previous studies shown in Table 3. The results show that the majority chemical composition of all ashes is silica dioxide *SiO_2_* (32.8%–84.2%) (Table 5). Sugarcane bagasse ash has much more *SiO_2_* while wood ash has less *SiO_2_* than coal fly ash Class C because some coarser quartz particles can adhere to the sugarcane bagasse surface and are harvested along with sugarcane bagasse [32].

**Table 5:** Chemical composition of biomass ashes and coal fly ash Class C.

Constituents	Coal Fly Ash Class C	Wood Ash	Sugarcane Bagasse Ash
SiO_2_	52.4	32.8	84.2
Al_2_O_3_	26.5	27.0	5.3
Fe_2_O_3_	7.5	2.2	2.9
CaO	8.7	11.7	2.0
MgO	3.0	9.1	1.0
SO_3_	1.9	-	-
Na_2_O	-	6.7	-
K_2_O	-	10.5	4.6

*Heavy Metal Test.* Total heavy metal concentrations were ascertained by the total constituent analysis and divided by 20 to compare with the maximum leachable concentration as shown by the US Environmental Protection Agency (EPA) (Table 6). The results show that all heavy metal concentrations are below the EPA maximum limit. If a waste is 100% solid, when the results of the total constituent analysis (heavy metal test) divided by 20 are less than the limits of the maximum leachable concentration, the EPA allows for a total constituent analysis instead of the toxicity characteristic leaching procedure (TCLP) extraction [33]. Thus, both sugarcane bagasse ash and wood ash can be used through replacement of soil to improve soil properties.

**Table 6:** Heavy metal contaminant concentration (ppm) in biomass ashes and coal fly ash Class C.

Heavy Metal	EPA	Sugarcane Bagasse Ash	Wood Ash	Fly ash Class C Type
Barium (Ba)	100	0.148	0.4608	1.72476
Arsenic (As)	5	0.00445	*bdl*	0.003935
Cadmium (Cd)	1	0.00185	*nd*	0.000845
Chromium (Cr)	*5*	0.0326	0.0328	0.036285
Lead (Pb)	5	*nd*	0.0116	0.010215
Mercury (Hg)	0.2	*nd*	*nd*	*nd*
Selenium (Se)	1	*nd*	*nd*	*nd*

Note: *bdl* = below detection limit; *nd* = non detect.

### 4.2. Hydraulic Conductivity

**Figure 5:**
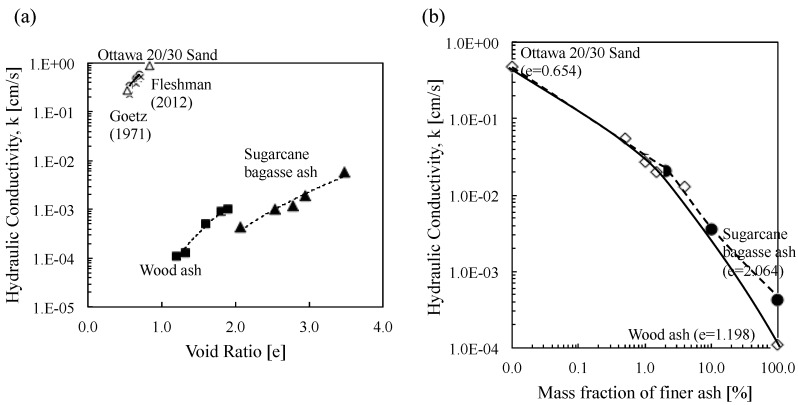
Hydraulic conductivity of tested materials: (**a**) variation of hydraulic conductivity of Ottawa 20/30 sand, wood and sugarcane ahses with void ratio; (**b**) hydraulic conductivity of Ottawa 20/30 sand-biomass ash mixutres as a function of fine ash fraction (0.5%~10%). Note the results of Ottawa 20/30 sand are comparable with previous test results [34,35].

Ottawa 20/30 sand shows a greater hydraulic conductivity (*K*) than biomass ashes, as anticipated, because of its larger particle size and pore size (Figure 5a). Additionally, wood ash with *d*_50_ = 0.180 shows greater hydraulic conductivity (*K*) than sugarcane baggage ash with *d*_50_ = 0.055 at a given void ratio. It is also observed that hydraulic conductivity (*K*) of all tested materials increases in line with the increase in void ratio reflecting increased number of channels for water flow (Figure 5a). More notably, in line with the increase in biomass ash contents, hydraulic conductivity of Ottawa 20/30 remarkably decreases (Figure 5b) because the smaller particles of biomass ashes fill in the pore space between large sand particles.

### 4.3. Consolidation

**Figure 6:**
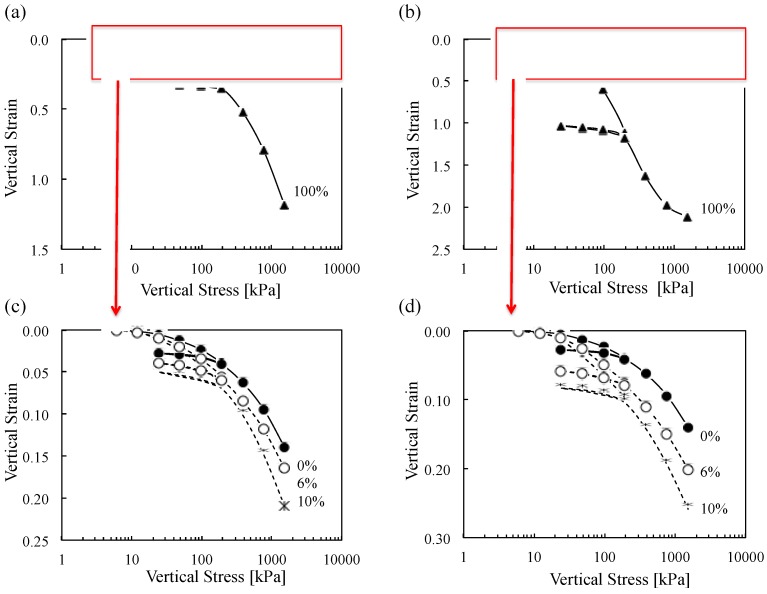
Vertical strain as a function of vertical effective stress in oedometer cell (**a**) Ottawa sand 20/30 containing wood ash (0%–100%), (**b**) Ottawa sand 20/30 containing sugarcane bagasse ash (0%–100%), (**c**) Ottawa sand 20/30 containing 0%–10% wood ash, (**d**) Ottawa sand 20/30 containing 0%–10% sugarcane bagasse ash.

**Figure 7:**
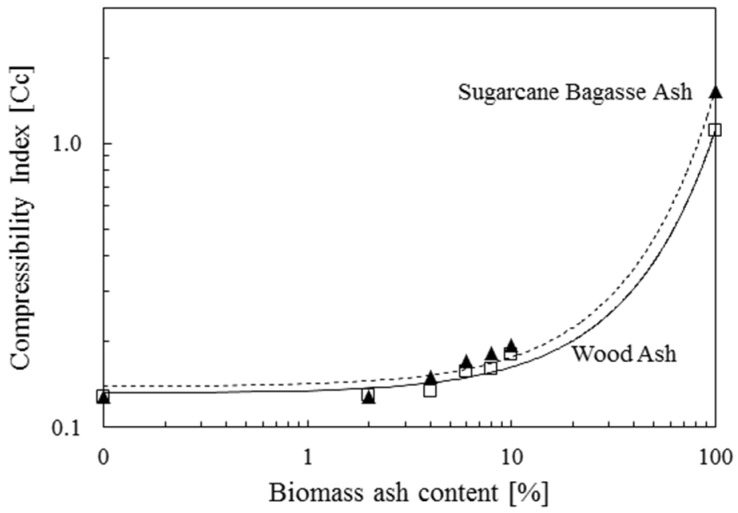
Compressibility Index of Ottawa 20/30 containing wood ash and sugarcane bagasse ash.

Figure 6 shows the vertical strain of Ottawa 20/30 sand, biomass ashes and Ottawa 20/30 sand-biomass ash mixtures as a function of vertical effective stress. Biomass ashes experience more vertical strain than Ottawa 20/30 sand and the mixture containing 2%~10% biomass ash. Sugarcane bagasse ash shows more vertical strain than wood ash. Particle shape, including angularity and roughness, affects compressibility under the condition of vertical strain loading. Analysis of the compressibility of tested materials revealed that compression index (*C_c_*) = 0.128 for Ottawa 20/30 sand, *C_c_* = 1.105 for wood ash and *C_c_* = 1.521 for sugarcane bagasse ash (Figure 7) reflecting the increase in compressibility of mixtures in line with an increase in biomass ash contents (note: Compression index (*C_c_*) is defined by the variation of the void ratio as a function of the change of effective stress in the logarithmic scale). Additionally, swelling indexes (*C_s_*), calculated from the unloading phase, are *C_s_* = 0.008 for Ottawa 20/30 sand, *C_s_* = 0.038 for wood ash and *C_s_* = 0.100 for sugarcane bagasse ash.

### 4.4. Shear Wave Velocity

**Figure 8:**
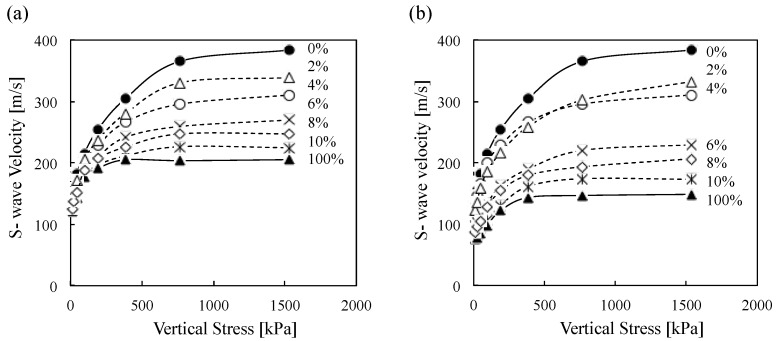
Shear wave velocity as a function of vertical effective stress (**a**) wood ash and (**b**) sugarcane bagasse ash.

The measured shear wave velocity (*V_s_*) of tested materials increases in line with an increase in applied vertical stress which is attributable to the development of better contact between the particles (*i.e*., increased contact area and coordination number, Figure 8). Previous studies show that shear wave velocity (*V_s_*) of the soils is the power function of applied stress [36,37]. Development of S-wave velocity *versus* vertical effective stress during odometer loading is illustrated in Figure 8 for all materials used in this study. S-wave velocity dramatically increases with the applied pressure range of 0–766 kPa and then slowly increases in the range of 766–1532 kPa (Figure 8). The results demonstrate that clean Ottawa 20/30 sand shows the highest shear wave velocity (*V_s_*)*.* Also, the measured velocities decrease in line with the increase in biomass ash contents.

## 5. Analysis and Discussion

### 5.1. Hydraulic Conductivity 

The lower hydraulic conductivity of biomass ashes (Figure 5a) can be explained according to the following: (1) Smaller particles of biomass ashes (*i.e*., the particle sizes of 78% of sugarcane bagasse ash and 25% of wood ash are less than 75-μm as illustrated in Figure 3) mean a smaller pore size [38] causing lower hydraulic conductivity. (2) A plate-like particle shape of biomass ashes and the alignment of those particles in a certain direction, causing a phenomenon called tortuosity, lead to an irregular flow path [38]. (3) Biomass ashes can absorb and hold much more water than sand due to the hydrophilicity, increasing the water uptake that causes hydraulic conductivity to become lower than Ottawa 20/30 sand [21].

**Figure 9:**
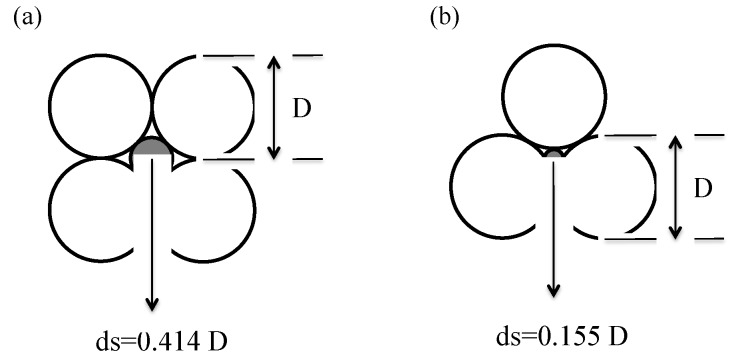
Theoretical pore sizes of soil packing: (**a**) the loosest tetrahedral packing, (**b**) the densest cubic packing.

In the Ottawa 20/30 sand-biomass ash mixtures, the smaller ash particles fill in the pore spaces between Ottawa 20/30 sands causing the decrease in hydraulic conductivity in line with an increase in biomass ash contents (Figure 5b). Theoretically, the pore size of the loosest tetrahedral packing is 0.414 *D* (Figure 9a) and the pore size of the densest cubic packing is 0.155 *D* (Figure 9b, Note, *D* is the diameter of host particle). Equation (1) shows the theoretical void ratio of Ottawa-biomass ash mixtures. Given the initial void ratio (*e* = 0.654) of mixtures used in this study, the mixtures are closed to ensure the loosest packing because the range of void ratios of Ottawa sand are *e* = 0.49–0.79 [39]. Most particle sizes of wood ash (*d_60_* = 0.260) and sugarcane bagasse ash (*d_60_* = 0.063) are less than 0.414 *D* (*i.e.*, 0.414 *D_10_* = 0.2691 and 0.414 *D_60_* = 0.3726). Thus, most ash particles can fill in the pore spaces in Ottawa 20/30 sands. Also, it is notable that a very low hydraulic conductivity can be obtained by adding very small amounts (only 1%–2% weights) of biomass ashes because the low specific gravity of biomass ashes lower the void ratio of Ottawa 20/30 sand-biomass ash mixture (Equation (1)). Additionally, the calcium ions in wood ash react with the silica or alumina in soil that leads to the formation of insoluble calcium silicates or aluminates, or both. Consequently, they can obstruct the water flow through the soil voids [40] and lead to the decrease in hydraulic conductivity along with the increase in biomass ash contents. In previous studies, Osinubi (2011) observed that hydraulic conductivity of soils treated with sugarcane bagasse ash decrease from 4.2 × 10^−10^ to 9.72 × 10^−11^ with 8% ash [40]. Also, previous laboratory tests show that foundry waste sand treated with a small amount of sugarcane bagasse ash reduces the hydraulic conductivity [41].

(1) $$e_{mix}=\frac{e_{sand}-x\;\left(\frac{Gs_{sand}}{Gs_{ash}}-1\right)}{1+x\;\left(\frac{Gs_{sand}}{Gs_{ash}}-1\right)}$$

where, *e_mix_* is the void ratio of sand-biomass ash mixture, *e_sand_* is the void ratio of sand, *Gs_sand_* is the specific gravity of sand, *Gs_ash_* is the specific gravity of biomass ash and *x* is replacement weight ratio between sand and biomass ash.

### 5.2. Constrained Modulus

**Figure 10:**
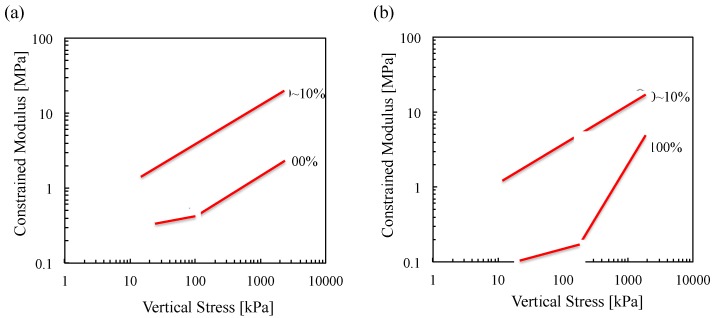
Constrained modulus as a function of vertical effective stress for all Ottawa 20/30 sand–biomass ash mixtures. Constrained modulus is calculated by stress increment in a way of dividing the average stress by the strain measured during load increment: (**a**) wood ash and (**b**) sugarcane bagasse ash.

The gradient of the stress-strain curve in Figure 6 is calculated by load increment to determine the development of the constrained modulus (*M*) with vertical stress. The summary plot in Figure 10 shows the increase in stiffness in line with increasing load, and decrease in stiffness in line with increasing biomass ash content. The shell structure of low-density wood and sugarcane bagasse ashes causes lower constrained moduli of biomass ashes that is consistent with previous results [42]. Also, the constrained moduli of wood and sugarcane bagasse ashes increase after reaching 100 and 200 kPa, respectively, because their shell structure may possibly be ruptured under the given pressures (Figure 10).

**Figure 11:**
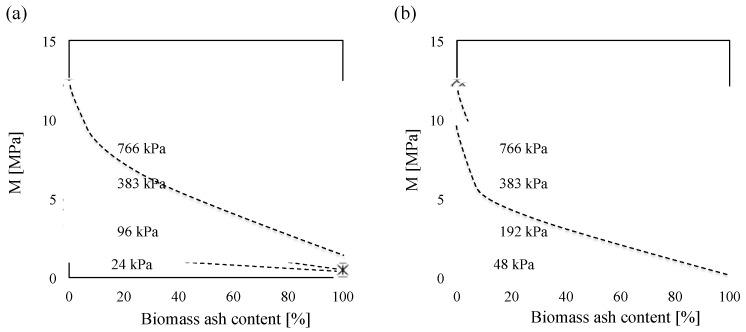
Constrained modulus variation with ash content: (**a**) wood ash and (**b**) sugarcane bagasse ash.

Constrained modulus of biomass ash decreases in line with increasing ash contents in mixtures (Figure 11).The mixtures containing up to 10% biomass ash content have highly constrained moduli. Because all tests are conducted at relatively low biomass ash content (<10%), it could be assumed that large sand particles should make the structural skeleton, while small biomass ash particles are generally placed in the pore space among large particles as shown in Figure 9. Therefore, the applied stress will be mainly transferred through the contact of large sand particles, and the contact of large particles will determine the constrained moduli of soils containing up to 10% biomass ashes.

### 5.3. Shear Modulus

The maximum shear modulus (*G_max_*) can be calculated using Equation (2).

(2) $$G_{max}=\text{ρ}\cdot V_{s}^{2}$$

where, ρ is the density of the soil specimen and *V_s_* is the S-wave velocity.

**Figure 12:**
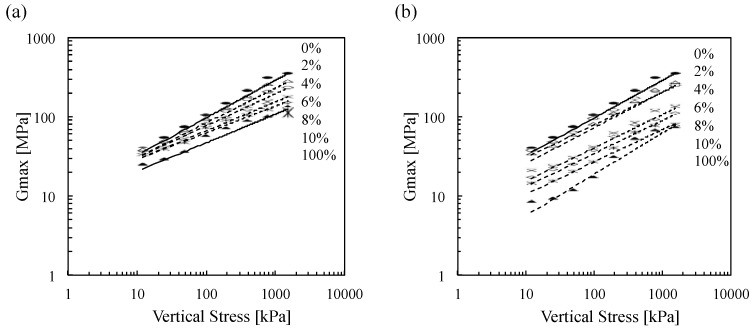
*G_max_* as a function of vertical effective stress during loading for all tested Ottawa 20/30 sand–biomass ash mixtures. The numbers in the figure denote the ash content in the mixture: (**a**) wood ash and (**b**) sugarcane bagasse ash.

Figure 12 is the summary plot of maximum shear modulus *versus* vertical effective stress while loading all tested Ottawa 20/30 sand-biomass ash mixtures. The results show that (1) the maximum shear modulus increases along with applied stress, (2) the maximum shear modulus of Ottawa 20/30 sand is higher than biomass ashes, and (3) the maximum shear modulus decreases along with biomass ash content.

**Figure 13:**
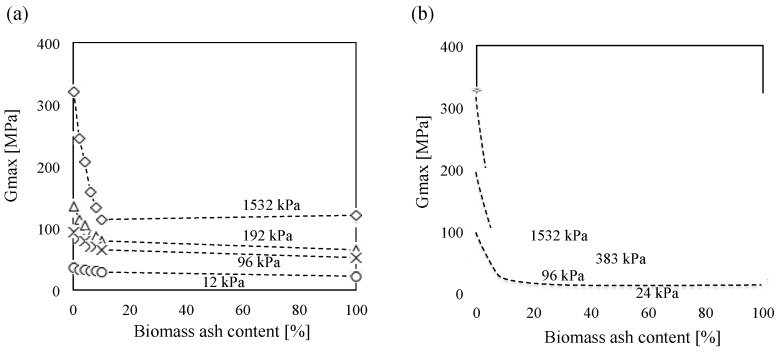
Small-strain shear modulus variation with ash contents: (**a**) wood ash and (**b**) sugarcane bagasse ash.

Figure 13 clearly demonstrates that the maximum shear modulus of clean Ottawa 20/30 sand dramatically decreased in line with the addition of biomass ashes. Previous studies on mixtures of large sand particles with small silt particles also show the decrease in *G_max_* (or *V_s_*) along with an increase in contents of small silt particles [43,44]. Because all tests are conducted with a relatively low biomass ash content (<10%), it could be assumed that large sand particles should make the structural skeleton, while small biomass ash particles are generally located in the pore space among the large particles. Therefore, applied stress will be mainly transferred through the contact of large sand particles, and the contact of large particles will determine the stiffness of the soils. Consequently, an increase in small biomass ash particles will disrupt the direct contact among large sand particles, leading to the decrease in maximum shear modulus in line with the increase in biomass ash content.

### 5.4. S-wave velocity and empirical relations

**Figure 14:**
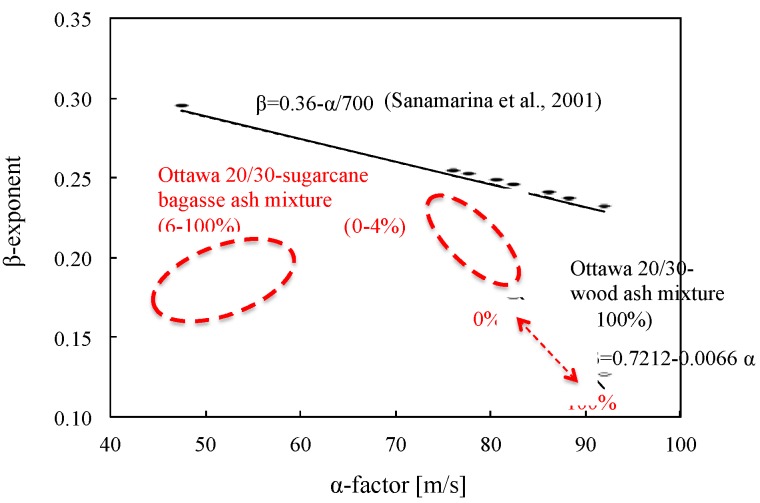
Variation of the α-factor and β-exponent used to model *G_max_*= α (σ*_o_’*/kPa)^β^.

S-wave velocity *V_s_* can be controlled by the nature of interparticle contacts and coordination. The effective stress governs the *V_s_* of uncemented particulate materials as predicted by the semi-empirical power relation [37] (Equation (3)):

(3) $$V_{s}=\text{α}{\left(\frac{{\text{σ}}_{o}^{'}}{1kPa}\right)}^{\text{β}}$$

where, σ*_o_’* is the average effective stress, and α and β are experimentally determined parameters. α-factor is the value of *V_s_* when σ*_o_’* = 1 kPa and it is related to packing, properties of the particles, contact behavior and fabric changes; β-exponent captures the sensitivity of *V_s_* to stress changes [37]. As the shear wave velocity is measured at different stress states, the β-exponent reflects not only contact behavior but fabric changes [37]. Generally, the higher the stiffness of the soils, the greater the α-factor but β-exponent decreases according to the following Equation (4) (Figure 14) [37].

(4) $$\text{β}=0.36-\frac{\text{α}}{700}$$

In the case of the sand-wood ash mixtures, there is a more pronounced decrease in the β-factor with the wood ash content when α-factor increases, indicating that the stiffness of the soil-wood ash mixture decreases with the addition of wood ash particles because 100% wood ashes have lower stiffness than Ottawa 20/30 sand. Furthermore, new relations between α and β parameters can be determined using the following Equation (5).

(5) β=0.7212−0.0066α

However, in the case of sugarcane bagasse ashes, α and β parameters follow the similar relations as Equation (5) with lower sugarcane bagasse ashes content (0%–4%) However, β factors rarely show constant values with higher sugarcane bagasse ashes content (6%–100%). Thus, the results show that more than 6% sugarcane bagasse ash in mixtures causes the changes in contact behavior as well as a fabric change in mixture that is consistent with the results in Figure 8.

## 6. Conclusions

The characteristics and engineering behaviors of wood and sugarcane bagasse ashes are evaluated in terms of hydraulic conductivity, consolidation, and S-wave velocity. The conclusions obtained from this study include the following:

- Photomicrographs taken with SEM show that both wood ash and sugarcane bagasse ash consist of a heterogeneous mixture of different particle sizes and shapes and have a shell structure that lowers their densities. Thus, the specific gravities of wood ash (*Gs* = 2.41), sugarcane bagasse ash (*Gs* = 2.34) and coal fly ash Class C (*Gs* = 2.58) are less than Ottawa 20/30 sand (*Gs* = 2.65). Also, their shell structure results in a higher consolidation index (*C_c_* = 1.105 for wood ash, *C_c_* = 1.521 for sugarcane bagasse ash) than Ottawa 20/30 sand (*C_c_* = 0.128) and a lower constrained modulus. However, the constrained moduli of wood and sugarcane bagasse ashes increase after reaching 100 and 200 kPa, respectively, because their shell structure may possibly rupture under the given pressures.
- XRD analysis displays that both wood ash and sugarcane bagasse ash mainly consist of *SiO_2_*, *Al_2_O_3_*, *Fe_2_O_3_*, *MgO*, *K_2_O* and *CaO* that are similar to coal fly ash Class C. The composition which takes up the greater portion of biomass ashes is the silicon dioxide (32.8%~84.2%, *SiO_2_*) that is responsible for the hydrophilicity and can lower hydraulic conductivity by absorbing more water.
- Wood and sugarcane bagasse ashes represent high alkalinity. Wood ash has a pH of 12.57 and sugarcane bagasse ash has a pH of 8.65, while Ottawa 20/30 sand is acidic indicating a pH of 4.01.
- Particle sizes of wood ash (*d_50_* = 0.180 mm) and sugarcane bagasse ash (*d_50_* = 0.055 mm) are about halfway between coal fly ash Class C (*d_50_* = 0.022 mm) and Ottawa 20/30 sand (*d_50_* = 0.870 mm). These smaller biomass ash particles can fill in the pore spaces between host particles of Ottawa 20/30 sand, which lowers the void ratio of Ottawa 20/30 sand–biomass ash mixtures. Thus, hydraulic conductivity values decrease significantly when replacing the soils with only 1%–2% of biomass ash. Thus, the Ottawa 20/30 sand–biomass ash mixtures can be used to decrease the hydraulic conductivity of geotechnical structures.
- Constrained modulus and shear modulus increase in line with increasing load but decrease in line with increasing biomass ash content. Ottawa 20/30 sand has higher constrained modulus and shear modulus than biomass ash. Ottawa 20/30 sand–biomass ash mixtures containing up to 10% ash can possess a high stiffness like Ottawa 20/30 sand because smaller biomass ash particles can be placed in the pore spaces between large sand particles, and larger sand particles can form the structural skeleton. Thus, the applied stress should be transferred mainly through the contact between large sand particles, and the contact between large particles will determine the stiffness of the soils.
- β-factor decreases in line with increasing wood ash content when the α-factor increases. However, in case of sugarcane bagasse ashes, α and β parameters follow similar relations as Equation (5) with lower sugarcane bagasse ash content (0%–4%). However, β factors rarely show constant values with higher sugarcane bagasse ash content (6%–100%).

## References

[1] Athanasopoulou A. (2013). Addition of lime and fly ash to improve highway subgrade soils. J. Mater. Civil Eng..

[2] Sumer M. (2012). Compressive strength and sulfate resistance properties of concretes containing Class F and Class C fly ashes. Constr. Build. Mater..

[3] Tastan E.O., Edil T.B., Benson C.H., Aydilek A.H. (2011). Stabilization of organic soils with fly ash. J. Geotech. Geoenviron. Eng..

[4] Kumar A., Walia B.S., Bajaj A. (2007). Influence of fly ash, lime, and polyester fibers on compaction and strength properties of expansive soil. J. Mater. Civil Eng..

[5] Field C.B., Campbell J.E., Lobell D.B. (2008). Biomass energy: The scale of the potential resource. Trends Ecol. Evolut..

[6] U.S. Department of Energy U.S. Billion-Ton Update: Biomass Supply for a Bioenergy and Bioproducts Industry. http://www1.eere.energy.gov/bioenergy/pdfs/billion_ton_update.pdf.

[7] Clarke S., Preto F. Biomass burn characteristics. http://www.omafra.gov.on.ca/english/engineer/facts/11-033.pdf.

[8] Udoeyo F.F., Inyang H., Young D.T., Oparadu E.E. (2006). Potential of wood waste ash as an additive in concrete. J. Mater. Civil Eng..

[9] Chusilp N., Jaturapitakkul C., Kiattikomol K. (2009). Utilization of bagasse ash as a pozzolanic material in concrete. Constr. Build. Mater..

[10] Edeh J.E., Agbede I.O., Tyoyila A. (2013). Evaluation of sawdust ash stabilized lateritic soil as highway pavement material. J. Mater. Civil Eng..

[11] Chauhan M., Mittal S., Mohanty B. (2008). Performance evaluation of silty sand subgrade reinforced with fly ash and fibre. Geotext. Geomembr..

[12] Dutta R.K. (2008). Effect of cement on the engineering properties of sand: A comparative study. Road Mater. Pavement Des..

[13] Milbrandt A. (2005). A Geographic Perspective on the Current Biomass Resource Availability in the United States.

[14] Huntrods D., Koundinya V. Sugarcane Profile. http://www.agmrc.org/commodities__products/grains__oilseeds/sugarcane-profile/.

[15] U.S. Department of Agriculture (USDA) The Economic Feasibility of Ethanol Production from Sugar in the United States. http://www.fsa.usda.gov/Internet/FSA_File/ethanol_fromsugar_july06.pdf.

[16] Genty T., Bussière B., Benzaazoua M., Zagury G.J. (2012). Capacity of wood ash filters to remove iron from acid mine drainage: Assessment of Retention mechanism. Mine Water Environ..

[17] Etiégni L., Campbell A.G. (1991). Physical and chemical characteristics of wood ash. Bioresour. Technol..

[18] Naik T.R., Kraus R.N., Siddique R. Demonstration of manufacturing technology for concrete and CLSM utilizing wood ash from Wisconsin. http://www4.uwm.edu/cbu/abstracts/04-551.pdf.

[19] Ganesan K., Rajagopal K., Thangavel K. (2007). Evaluation of bagasse ash as supplementary cementitious material. Cem. Concr. Compos..

[20] Guo Y., Zhao C., Chen X., Li C. (2015). CO_2_ capture and sorbent regeneration performances of some wood ash materials. Appli. Energy.

[21] Amin N. (2010). Use of bagasse ash in concrete and its impact on the strength and chloride resistivity. J. Mater. Civil Eng..

[22] Kutchko B.G., Kim A.G. (2006). Fly ash characterization by SEM–EDS. Fuel.

[23] Misra M.K., Ragland K.W., Baker A.J. (1993). Wood ash composition as a function of furnace temperature. Biomass Bioenergy.

[24] Grzeszczyk S., Lipowski G. (1997). Effect of content and particle size distribution of high-calcium fly ash on the rheological properties of cement pastes. Cement Concr. Res..

[25] Abdullahi M. (2006). Characteristics of wood ash/OPC concrete. Leonardo Electron. J. Pract. Technol..

[26] Teixeira S.R., de Souza A.E., de Almeida Santos G.T., Vilche Peña A.F., Miguel A.G. (2008). Sugarcane bagasse ash as a potential quartz replacement in red ceramic. J. Am. Ceram. Soc..

[27] American Coal Ash Association (1995). Fly Ash Facts for Highway Engineers.

[28] Lee J.S., Santamarina J.C. (2005). Bender elements: Performance and signal interpretation. J. Geotech. Geoenviron. Eng..

[29] Fernandez A.L. (2000). Tomographic Imaging the State of Stress. Ph.D. Thesis.

[30] Geliga E.A., Ismail D.S.A. (2010). Geotechnical properties of fly ash and its application on soft soil stabilization. UNIMAS E J. Civil Eng..

[31] Ban C.C., Ramli M. (2011). The implementation of wood waste ash as a partial cement replacement material in the production of structural grade concrete and mortar: An overview. Resour. Conserbation Recycl..

[32] Cordeiro G.C., Filho R.D., de Almeida R.S. (2011). Influence of ultrafine wet grinding on pozzolanic activity of submicrometre sugar cane bagasse ash. Adv. Appli.Ceram..

[33] Goetz R.O. (1971). Investigation into Using Air in the Permeability Testing of Granular Soils.

[34] Method 1113 Toxicity Characteristic Leaching Procedure. http://www3.epa.gov/epawaste/hazard/testmethods/sw846/pdfs/1311.pdf.

[35] Fleshman M.S. (2012). Laboratory Modeling of Critical Hydraulic Conditions for the Initiation of Piping. Marster’s Thesis.

[36] Roesler S.K. (1979). Anisotropic shear modulus due to stress anisotropy. J. Geotech. Eng. Div..

[37] Santamarina J.C., Klein K., Fam M. (2001). Soils and waves: Particulate materials behavior, characterization and process monitoring. J. Soil Sedim..

[38] Murray E.J., Jones R.H., Rix D.W. Relative Importance of Factors Influencing the Permeability of Clay Soils. http://www.murrayrix.co.uk/murrayrix/PERMEABILITY.pdf.

[39] Hough B.K. (1957). Basic Soils Engineering.

[40] Osinubi K., Moses G., Han J., Alzamora D.E. (2011). Compacted Foundry Sand Treated with Bagasse Ash As Hydraulic Barrier Material. Geo-Frontiers 2011.

[41] Kolawole J., Osinubi K., Adrian O., Ebemeru A. (2013). Hydraulic conductivity of compacted lateritic soil treated with bagasse ash. Int. J. Environ. Waste Manag..

[42] Anshits N.N., Mikhailova O.A., Salanov A.N., Anshits A.G. (2010). Chemical composition and structure of the shell of fly ash non-perforated cenospheres produced from the combustion of the Kuznetsk coal (Russia). Fuel.

[43] Salgado R., Bandini P., Karim A. (2000). Shear strength and stiffness of silty sand. J. Geotech. Geoenviron. Eng..

[44] Iwasaki T., Tatsuoka F. Dynamic soil properties with emphasis on comparison of laboratory tests and field measurements. http://www.iitk.ac.in/nicee/wcee/article/6_vol3_2303.pdf.

